# Resuscitating the Dying Autopsy

**DOI:** 10.1371/journal.pmed.1001927

**Published:** 2016-01-12

**Authors:** Quique Bassat, Paola Castillo, Pedro L. Alonso, Jaume Ordi, Clara Menéndez

**Affiliations:** 1 ISGlobal, Barcelona Ctr. Int. Health Res. (CRESIB), Hospital Clínic, Universitat de Barcelona, Barcelona, Spain; 2 Centro de Investigação em Saúde de Manhiça (CISM), Maputo, Mozambique; 3 Department of Pathology, Hospital Clinic, Universitat de Barcelona, Barcelona, Spain; 4 Global Malaria Program, World Health Organisation, Geneva, Switzerland

## Abstract

Clara Menéndez and colleagues consider the importance of conducting autopsies and possible alternative methods for determining cause of death in resource-limited settings.

**Summary Points**

- Despite the relevant information that postmortem examinations can provide, autopsy rates have been declining worldwide in the last few decades.
- Autopsies are especially necessary in low-income countries, where reliable information on cause of death is much more limited and in vivo clinical diagnosis is hampered by the scarce availability of diagnostic tools.
- The use of the autopsy or of robust substitutes for this procedure needs to be encouraged as a mechanism for the continuous improvement of clinical diagnosis and as a complement for cause-of-death investigation and surveillance.

## Introduction

Why did the patient die? Throughout the history of medicine, the answer to this key question has often remained packed with uncertainties. These uncertainties can be generally resolved by conducting an autopsy. Autopsy, from the Greek autopsia (*αUTOψία*), literally means “seeing for one’s self” and refers to the dissection of the dead body to determine the cause of death (CoD) through the observation, following a systematic approach, of any macro- or microscopic pathological changes that illnesses produce in human beings [1]; other terms utilized include “postmortem examination” or “necropsy.” In this essay, we discuss the past, present, and future perspectives of clinical autopsies.

## The Declining Rates in Autopsy Practice

Although autopsies have been conducted for centuries [2], their golden age can be traced back to the end of the 19th century, when autopsies moved out from anatomy theatres and private homes to hospitals and public mortuaries. In hospitals, autopsies evolved into a routine component of clinical practice, and the proportion of in-hospital deaths subjected to an autopsy was considered as an indicator of the quality of the hospital [1]. By the beginning of the 1960s, autopsy rates started decreasing in the majority of rich countries [3–7], and the trend continues to this date. This decline was probably associated with the improvements in premortem clinical diagnosis that occurred at that time. From the clinician’s perspective, the increasing availability of a plethora of in vivo imaging and laboratory procedures [8,9] may have demoted the diagnostic potential of postmortem examinations, which would appear less necessary, a perception perhaps also shared by family members supposed to provide consent [10,11]. In this context, clinicians may also be reluctant or feel unqualified to undergo a burdensome consent process with the family of the deceased [12] or feel threatened by the auditing capacity of autopsies, which could reveal a previous medical and/or surgical malpractice [13,14].

### The Importance of Post-mortem Examinations

In spite of the advances in diagnostic methods in recent decades, the autopsy, if performed well, remains, by consensus, the gold standard methodology for diagnosing most medical conditions [14]. Antemortem diagnostic errors, detected during the autopsy as clinical-pathological discrepancies, are relatively frequent [15–17] and highlight the importance of this procedure in establishing the ultimate CoD. These diagnostic inaccuracies are considered to occur at even higher rates in low-income countries, where premortem diagnostic support is limited [18,19].

Autopsies have been, and still remain, an indispensable research tool. Many advances in medicine have been possible through postmortem methods, including the investigation of emerging infections, genetic or metabolic diseases, or transplant-associated lesions. The routine practice of autopsies offers also an important epidemiological window to the health status of populations. As an example, autopsies conducted in theoretically healthy young soldiers dying from injuries incurred during armed conflicts have confirmed an alarmingly high rate of atherosclerotic changes in the coronary arteries, long before ischemic heart disease becomes clinically apparent [20]. Finally, autopsies are also important for the mourning families, who may find comfort after understanding what killed their beloved one and what can be done to prevent similar deaths and after being reassured that the clinical care provided was appropriate [3,11].

### Autopsies Are Especially Necessary in Low-Income Countries

All of the aforementioned arguments supporting the need to foster postmortem examinations are valid for low-income countries (LICs). In these settings, where vital registration systems are fragile [21], access to health is uncertain, and morbidity surveillance is generally weak, autopsy data could potentially contribute to completing the CoD picture and provide very actionable information for health planning and prioritisation [22–24], at least for those preventable deaths affecting the most vulnerable populations. However, the feasibility of conducting autopsies in LICs for investigating causes of death faces notable barriers, including, among others, the lack of pathology expertise and infrastructures, the fact that many deaths occur outside of the health system [23], and cultural and/or religious apprehension leading to a poor acceptability of the traditional and highly disfiguring autopsy procedure [10,25]. The recognition of the significant public health value of conducting CoD surveillance in these settings has led to an increasing interest in developing alternatives to the autopsy that are acceptable at the community level but capable of providing robust aetiological data.

## Alternatives for Settings Where the Autopsy Is Unacceptable

The WHO-recommended verbal autopsy (VA) tool is currently being utilized in resource-constrained settings for CoD surveillance [26]. VA implies conducting a standardized interview with the family of the deceased and interpreting the data gathered in order to infer a possible CoD [3]. VAs can be done long after death and are well accepted, but they have limited accuracy [27,28]. Initial validation studies in Africa showed that VAs were generally valid to get accurate information on the cause of children’s severe illnesses [29], and this led to their expansion in LICs as a means of determining CoD. However, although VAs can be done long after death and are well accepted, they have lately been shown to be less accurate than expected, especially for conditions with low specificity, such as those leading to perinatal and maternal deaths [27,28]. Current estimates of CoD from LICs rely on VAs or on clinical diagnoses, but both methods are prone to misclassification errors. Thus, knowledge of the main causes of death in those regions is rather poor, which is a critical limitation for prioritizing effective health programs and evaluating their impact [23,24]. Besides the VA, innovative strategies have been developed [30] for places where autopsies may be hampered by low acceptability. These methods should ideally provide results as similar to the conventional autopsy as possible. Imaging-based methods using magnetic resonance imaging, a computerized scanner, or ultrasounds [31] have been proposed [32]. These methods offer advantages, including their noninvasive nature (this is the reason they are known as virtual autopsies or “virtopsies” [33]) and consequently are highly acceptable. However, their elevated costs and reliance on sophisticated equipment and skilled personnel are critical limitations for their generalized introduction, particularly in LICs [3]. The minimally invasive autopsy (MIA), which may be used as a complement to these imaging techniques or independently of them [34,35], may represent a robust alternative to the conventional autopsy, and our team has been working in the last three years in the validation of its performance against the complete autopsy for CoD investigation in LIC [36,37]. MIA includes a compendium of postmortem sampling of key organs using fine biopsy needles, aiming at obtaining tissue fragments and body fluids for a thorough investigation of the CoD. Its nondisfiguring nature, shorter duration, and technical simplicity, in addition to its higher level of safety, are additional advantages of the procedure. Moreover, MIA offers the possibility of assessing the samples obtained for the presence of microorganisms, something seldom possible in the conventional autopsy on account of the high contamination rate ensuing from the dissection of the body. Importantly, MIAs could also provide a set of invaluable validation data for other methods such as the VA, which will surely continue to play a major role for CoD surveillance in LICs, enhancing their diagnostic accuracy by improving data capture instruments and analytical algorithms for their interpretation, something that the conventional autopsies have not been able to provide until now. The easiness of the MIA could be an essential component of the “democratization” of the methodology and will be indispensable for a future implementation as a surveillance tool in LICs. Validation of its performance against the complete autopsy and acceptability and feasibility studies to inform on the appropriate and locally tailored prerequisites for its application in different geographical, cultural, and religious backgrounds are needed.

## Conclusions

The use of the autopsy or of robust substitutes for this procedure needs to be encouraged as a mechanism for the continuous improvement of clinical diagnosis and as a complement for CoD investigation and surveillance. While all the challenges facing the provision of autopsies in LICs cannot be immediately solved, methods such as the MIA could easily be implemented on a wider scale, coupled with programs to ensure the building of capacity for local pathologists; this would surely contribute to reducing the stigma that postmortem practices currently involve. In high-income countries, reappraisal of the role of the autopsy is needed, and this will require that both the general public and the medical community are resensitized about its individual potential and wider public health benefits.

## Abbreviations

CoD: cause of death
LIC: low-income country
MIA: minimally invasive autopsy
VA: verbal autopsy
